# Trends in osteoporotic fracture and related in-hospital complications during the COVID-19 pandemic in Alberta, Canada

**DOI:** 10.1007/s11657-022-01114-9

**Published:** 2022-08-03

**Authors:** T. Oliveira, D. L. Kendler, P. Schneider, A. G. Juby, R. J. Wani, M. Packalen, S. Avcil, S. Li, C. Waters-Banker, E. Graves, S. McMullen, J. Brown

**Affiliations:** 1 Amgen Canada Inc, Mississauga, ON Canada; 2 Department of Medicine, Division of Endocrinology, University of British Columbia, Vancouver, BC Canada; 3 Division of Orthopaedic Trauma, Department of Surgery, University of Calgary, Calgary, AB Canada; 4 Department of Medicine, Division of Geriatric Medicine, University of Alberta, Edmonton, AB Canada; 5 Medlior Health Outcomes Research Ltd, Suite 210 – 28 Quarry Park Blvd, Calgary, AB T2C 5P9 Canada; 6 Department of Medicine, Laval University and CHU de Québec Research Centre, Quebec City, QC Canada

**Keywords:** COVID-19, Pandemic, Osteoporosis, Fractures, In-hospital complications

## Abstract

**Summary:**

Fragility fractures (i.e., low-energy fractures) account for most fractures among older Canadians and are associated with significant increases in morbidity and mortality. Study results suggest that low-energy fracture rates (associated with surgical intervention and outcomes) declined slightly, but largely remained stable in the first few months of the COVID-19 pandemic.

**Purpose/introduction:**

This study describes rates of low-energy fractures, time-to-surgery, complications, and deaths post-surgery in patients with fractures during the coronavirus disease (COVID-19) pandemic in Alberta, Canada, compared to the three years prior.

**Methods:**

A repeated cross-sectional study was conducted using provincial-level administrative health data. Outcomes were assessed in 3-month periods in the 3 years preceding the COVID-19 pandemic and in the first two 3-month periods after restrictions were implemented. Patterns of fracture- and hospital-related outcomes over the control years (2017–2019) and COVID-19 restrictions periods (2020) were calculated.

**Results:**

Relative to the average from the control periods, there was a slight decrease in the absolute number of low-energy fractures (*n* = 4733 versus *n* = 4308) during the first COVID-19 period, followed by a slight rise in the second COVID-19 period (*n* = 4520 versus *n* = 4831). While the absolute number of patients with low-energy fractures receiving surgery within the same episode of care decreased slightly during the COVID-19 periods, the proportion receiving surgery and the proportion receiving surgery within 24 h of admission remained stable. Across all periods, hip fractures accounted for the majority of patients with low-energy fractures receiving surgery (range: 58.9–64.2%). Patients with complications following surgery and in-hospital deaths following fracture repair decreased slightly during the COVID-19 periods.

**Conclusions:**

These results suggest that low-energy fracture rates, associated surgeries, and surgical outcomes declined slightly, but largely remained stable in the first few months of the pandemic. Further investigation is warranted to explore patterns during subsequent COVID-19 waves when the healthcare system experienced severe strain.

**Supplementary Information:**

The online version contains supplementary material available at 10.1007/s11657-022-01114-9.

## Introduction

Fragility fractures (FF) due to osteoporosis account for more than 80% of all fractures in Canadians over the age of 50. The risk of a major osteoporotic fracture in Canada is among the highest in the world, with an annual incidence of 211,986, more than heart attacks, strokes, and breast cancer cases combined [1]. FF occur following low-energy trauma, which is defined as a fall from standing height or less (e.g., tripping, or transitioning out of bed), as opposed to high-energy trauma fractures, which can be experienced falling from greater than standing height or from high impact forces (e.g., motor vehicle accidents) [1]. Hip fractures are the most common FF and are associated with increased morbidity and mortality [2]. Hip FF are also associated with high medical costs, particularly within the first-year post-fracture, with the largest proportion of cost dedicated to hospitalization and long-term care (mean cost 1-year post-hip fracture CA$62,793 ± CA$44,438 in 2017) [2, 3]. In Canada, with the aging demographics, the annual economic cost of hip fracture alone is projected to reach $2.4 billion by 2041 [3]. However, first-year incremental healthcare costs of other FF such as the wrist (CA$16,541 ± CA$25,687), vertebrae (CA$40,900 ± CA$47,837), and pelvis (CA$45,350 ± CA$38,572) are substantial as well, and contribute to an overall estimated cost of FF across Canada in excess of CA$1.9 billion annually [2].

FF are associated with a significant increase in mortality, comparable to other chronic diseases like cardiovascular disease [4, 5]. Individuals who experience FF have a decreased 1-year and 5-year survival rates compared to non-fracture individuals (1-year survival: fracture 85.6% vs. non-fracture 94.7%; 5-year survival: fracture: 58.5% vs. non-fracture 75.2%) [5]. Due to the significant morbidity, mortality, and cost associated with FF, the Public Health Agency of Canada has recently highlighted osteoporosis and related fractures as a major public health concern [6].

The orthopedic management of FF requires significant resources and patient care. Outpatient care requires multiple visits to a fracture clinic, while inpatient care can include surgical intervention, post-surgical care, and management of complications. Surgical complications are reportedly found in up to 30% of cases, and require increased patient care [7]. Rates of complications are higher in older patients, who are more likely to experience FF, and additionally may have one or more comorbid conditions (e.g., diabetes, stroke, osteoarthritis), thus further complicating the treatment approach [8]. Elderly individuals with FF are more likely to require hospitalization for non-operative fractures as well, such as stable pelvic and humeral fractures [3].

The pandemic has impacted many conditions requiring admission to the emergency department (ED). For example, metropolitan hospitals in Italy reported highly significant decreases in atraumatic musculoskeletal issues, sport-related injuries, and motor vehicle accidents presenting to the ED during lockdown [9]. Despite the decrease reported for musculoskeletal injuries, Italian hospitals reported an increased rate of FF in the elderly population over the same lockdown period [9]. Conversely, in France, the absolute number of hip fractures in patients 50 years of age and older decreased by 11% during the first coronavirus-19 (COVID-19) lockdown period [10]. The impact of the pandemic on the incidence of FF and associated outcomes is anticipated to vary across jurisdictions due to variations in lockdown measures and public health policies or practices. General healthcare resources and resources that support fracture care (e.g., fracture liaison services) were disrupted and/or strained globally at various timepoints during the pandemic [11]. Logistical and operational changes to hospital organization (e.g., surgical prioritization, staffing modifications, bed reallocation) and pre-surgical protocols (e.g., COVID-19 testing), in addition to orthopedic ward and rehabilitation infection practices, have all reduced capacity for FF treatment [11–19]. It has also been hypothesized that the pandemic may have led to potential delays in timely care for certain conditions due to the fear of going to the ED. Delays in care experienced by Canadians suffering FF during the COVID-19 pandemic could have potentially exacerbated the already existing osteoporotic care gap and negatively impacted the socioeconomic burden of osteoporosis in Canada.

The purpose of this study is to characterize the extent to which osteoporosis care patterns were affected during the first 6 months of the pandemic, relative to a pre-pandemic control period, within a Canadian setting. The primary objective was to describe the overall rate of low-energy fractures occurring at several common osteoporotic fracture sites (e.g., hip, femur, vertebral) during the initial months (first wave) of the COVID-19 pandemic relative to rates in the 3 years prior. The secondary objectives were to describe time-to-surgery, and complications and deaths post-surgery in fracture patients during and 3 years prior to the pandemic.

## Methods

### Study design and population

**Fig. 1:**
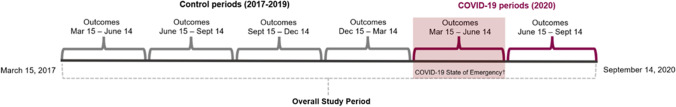
Study design and COVID-19^†^ lockdown overview. ^†^The COVID-19 State of Public Health Emergency in Alberta resulted in the temporary residential lockdown, closure/restricted access of public facilities, and cancellation of elective surgeries (i.e., lockdown period)

A repeated cross-sectional study design, using 3-month periods of population-level administrative health data from the province of Alberta, Canada, was used for this study (Fig. 1). Health records for patients aged 50 or older with a diagnosed fracture in the health system data were identified. Outcomes of interest included fracture events, fracture repair surgeries within 24 h of discharge from the ED (i.e., transfer of care), in-patient hospital length of stay (LOS), complications in the 30 days following surgery, and in-hospital all-cause mortality. Complications were reported as a composite indicator including pneumonia, post-surgical infections, pulmonary embolism, venous thromboembolism, and myocardial infarction.

Data for these events of interest were pulled from the Alberta Health administrative datasets, including the Discharge Abstract Database (DAD), the National Ambulatory Care Reporting System (NACRS), Practitioner’s Claims (Alberta’s physician billing system), and Population Registry, using the diagnostic codes from the International Classification of Disease, Ninth revision, Clinical Modification (ICD-9-CM), and the International Statistical Classification of Diseases and Related Health Problems, 10^th^ revision, Canada (ICD-10-CA).To facilitate access to timely information from the health system, this study was conducted using “open year” administrative health data that has not been prepared for research use via the additional review and validation that is implemented once the system closes.

Outcomes were assessed in 3-month periods for the 3 years before COVID-19 pandemic restrictions were implemented in Alberta (March 2017 to March 2020), defined as the control period, as well as for the first two periods following the implementation of COVID-19 pandemic restrictions (March 15 to September 15, 2020), defined as the COVID-19 period (Fig. 1). To provide context to the COVID-19 period, Alberta declared a local state of public health emergency on March 17, 2020, and on March 27, the province announced non-essential businesses would be temporarily closed, elective surgeries were canceled/postponed, and gatherings limited to 15 people (i.e., lockdown period). On May 25, 2020, the state of emergency was lifted, and Alberta initiated a “relaunch” program in all areas of the province.

### Data analysis

Outcomes were calculated for each cross-sectional period throughout the study period and the resulting descriptive results are reported as plots over time. A weighted average in the number of fracture events was used (calculated from each year of the control period), to allow for a descriptive comparison of the relative increase or decrease during the COVID-19 period. For the remaining outcomes, characterizations were presented year over year, with the COVID-19 period relative to the 2019 control year, to describe the annual variability observed during the control periods. Descriptive analyses were conducted between the same 3-month periods each year to account for seasonal variation. Changes between the control and COVID-19 periods were calculated as percentage point changes.

Individuals ≥ 50 years of age presenting to the ED who received a fracture diagnosis code were included in each cross-sectional period. Fractures were categorized as high-energy (ICD-10 codes for accidents, injuries involving multiple body regions, and falls) or low-energy fractures (all other fractures). Low-energy fractures were then further described by anatomical location and stratified by geographic region (Urban zones – Calgary and Edmonton; Rural zones – Central, North, and South), sex, and age (50–64 years, 65–79 years, ≥ 80 years).

Outcomes were reported as an absolute number and as a proportion of the overall fracture diagnosis code and/or the surgical repair population(s) depending on the outcome being reported. Patients with a low-energy fracture who received surgery within 24 h of discharge from the ED were reported as all fractures and hip fractures. Mortality for low-energy fracture was reported as surgical, and then further as all fractures and then specifically hip fractures. In compliance with privacy regulations, results representing less than 10 patients were not reported.

## Results

### Overall and low-energy fractures

**Fig. 2:**
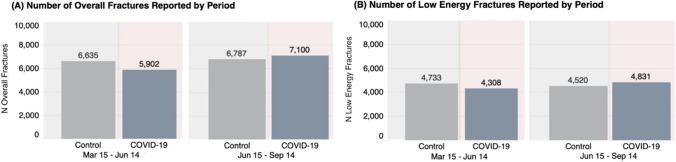
(**A**) Number of overall fractures reported by period. Data are presented as an average over the 3-year control period (grey) and absolute for the COVID-19 period* (red). (**B**) Low-energy^†^ fractures presented as an average for the control period (grey) and as absolute for theCOVID-19 period. Abbreviations: N: number; Mar: March; Jun: June; Sept: September; COVID-19: coronavirus disease 2019 SARS-CoV-2 virus. *The COVID-19 State of Public Health Emergency in Alberta resulted in the temporary residential lockdown, closure/restricted access of public facilities, and cancellation of elective surgeries (i.e., lockdown period). †Low-energy fractures are defined as fractures sustained when falling from standing height or less

To better understand any observed change in the COVID-19 period relative to the control period, overall fracture rates were analyzed to determine if there was a discernible difference in the number of new fractures presenting to the ED that may influence downstream outcomes. During the COVID-19 period, there was a slight decrease in the absolute number of overall fractures reported to the hospital in the first period (March to June 2020) relative to the weighted average number of overall fractures during the same period from the control years (Fig. 2). Similarly, there was a slight decrease in the number of low-energy fractures during the March to June COVID-19 period (Fig. 2).

A slight rise in overall and low-energy fractures was observed during the subsequent June to September (2020) COVID-19 period relative to the weighted average from the 3-year control period. Despite the slight fluctuations in absolute fractures, low-energy fractures represented approximately 68–73% of overall fractures during the COVID-19 period relative to 65–71% of overall fractures during the control period.

**Fig. 3:**
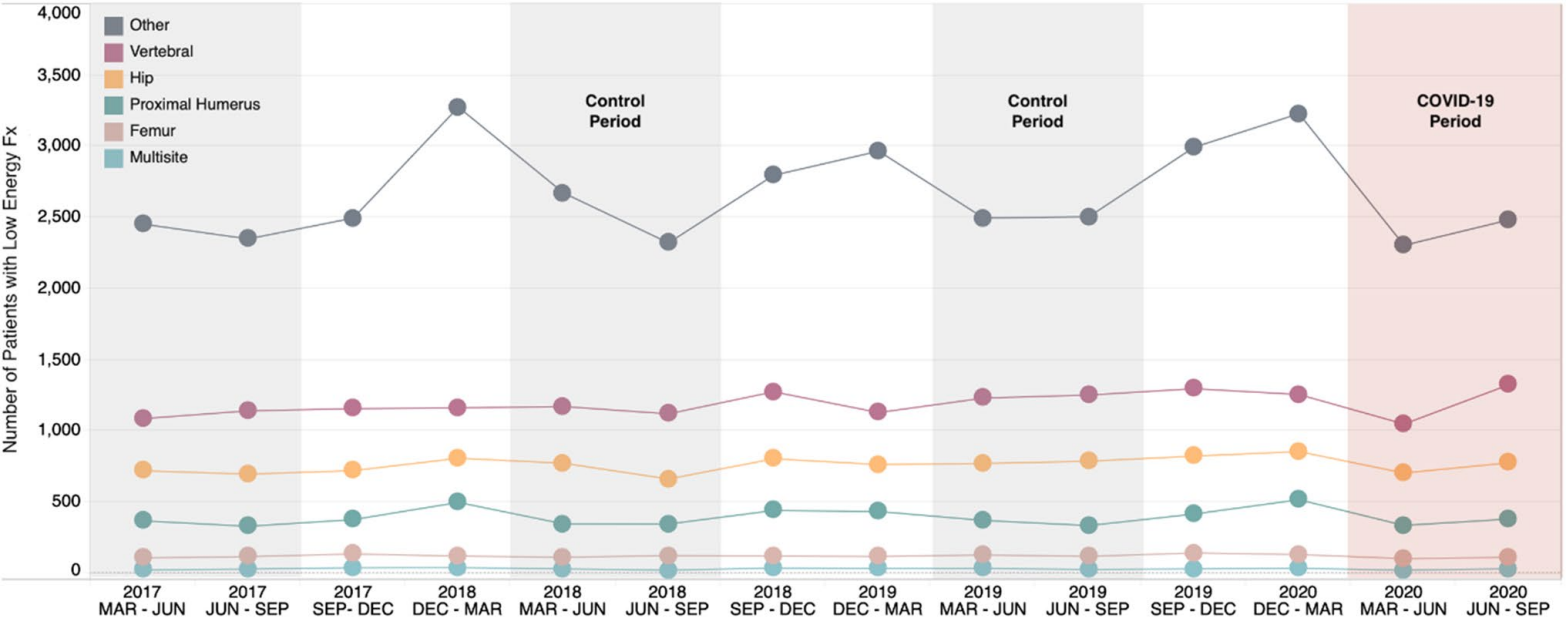
Number of patients experiencing low-energy fracture* in Alberta, Canada, from 2017 to 2020 stratified by fracture site. Note: The grey shaded areas represent the control periods evaluated in this study. The red shaded area represents the COVID-19 pandemic period where in the first 3 months (Mar–Jun) a COVID-19 State of Public Health Emergency in Alberta was active, which resulted in a temporary residential lockdown, the closure/restricted access of public facilities, and the cancellation of elective surgeries (i.e., lockdown period). Abbreviations: Fx: fracture; N: number; Mar: March; Jun: June; Sep: September; Dec: December. *Low-energy fractures are defined as fractures sustained when falling from standing height or less

In general, low-energy fracture rates for women remained approximately double, or greater, than fracture rates among males across the control and COVID-19 periods and all stratified age groups (Supplementary Table 1). Low-energy fracture rates remained relatively stable for both females and males across all stratified age groups in both COVID-19 periods, relative to the 2019 control period. The greatest reduction in low-energy fracture rates, when comparing the COVID-19 periods, was observed for females ≥ 80 years of age in the March to June COVID-19 period (*n* =  − 139) and males ≥ 80 years of age also in the March to June COVID-19 period (*n* =  − 93). Seasonal fluctuations in fracture rates were observed across the study period for some fracture locations. Low-energy fractures of the hip and vertebral and proximal/upper humerus dropped slightly in the first COVID-19 period, before slightly rising again in the second COVID-19 period (Fig. 3).

### Surgical intervention in less than 24 h of discharge from the ED

**Fig. 4:**
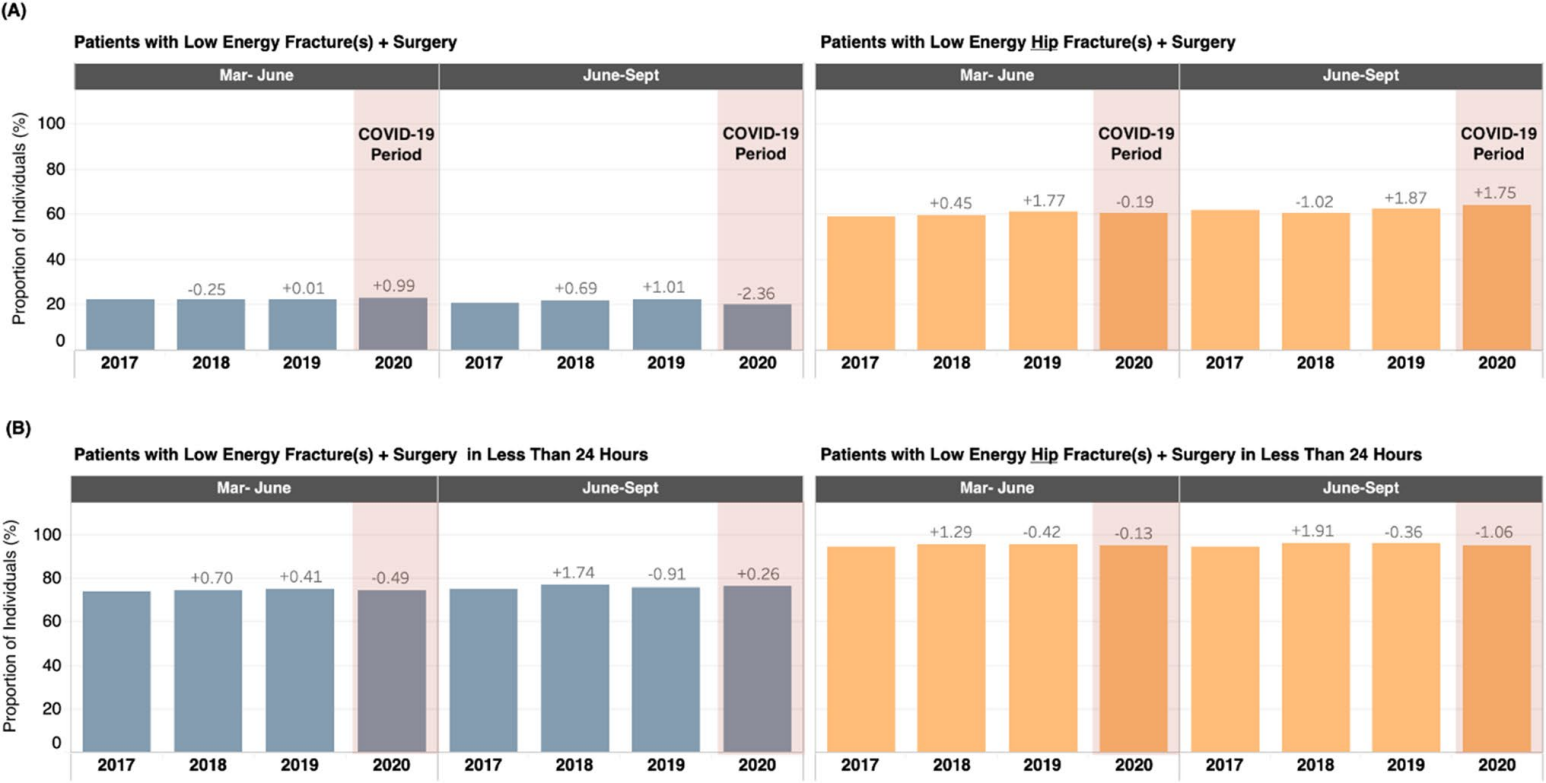
All low-energy fracture repair and surgical intervention in less than 24 h. (**A**) The absolute number and change in percentage points from the previous year of patients who received a fracture diagnosis and surgical fracture repair code within the same episode of care for all low-energy fractures* and low-energy hip fractures*. (**B**) Patients receiving fracture repair surgery within 24 h of discharge from the ED^†^ for all low-energy fractures* and low-energy hip fractures* in Alberta, Canada (2017–2020). Abbreviations: COVID-19: coronavirus disease 2019 SARS-CoV-2 virus; second period; Mar: March; Sep: September; ED: Emergency Department. *Low-energy fractures are defined as fractures sustained when falling from standing height or less. ^†^Data reported reflects the number of people diagnosed with a low-energy fracture who received surgical intervention within 24 h of discharge from the emergency department

The number of patients with a low-energy fracture and who received a fracture repair surgery within the same episode of care decreased slightly during the COVID-19 period relative to the 2019 control period (*n* =  − 64 people from March to June; *n* =  − 98 people from June to September). However, for the March to June period, this was a 1.0% point increase in the proportion of patients receiving surgery in the same episode of care among all patients presenting with a low trauma fracture relative to 2019, as the total number of patients presenting with low trauma fractures was slightly lower in this period (Fig. 4; Table 1).

**Table 1:** Absolute and percentage point changes for surgical intervention < 24 h, deaths, and complications for patients with low-energy* fracture diagnosis and surgical repair codes by study period in Alberta, Canada (2017–2020)

	2017		2018		Difference (2018–2017)		2019		Difference (2019–2018)		2020 COVID-19 lockdown^†^		Difference (2020–2019)	
	*n*	%	*n*	%	*n*	% point change^‡^	*n*	%	*n*	% point change^‡^	*n*	%	*n*	% point change^‡^
**Number of people with low trauma fracture(s)**														
Mar–June	4531	-	4884	-	353	-	4784	-	− 100	-	4308	-	476	-
June–Sept	4438	-	4361	-	− 77	-	4761	-	400	-	4831	-	70	-
**Number of people with low trauma Fx(s) + surgical Fx repair**														
Mar–June	1028	22.7	1096	22.4	68	− 0.3	1074	22.4	− 22	0.0	1010	23.4	− 64	1.0
June–Sept	932	21.0	946	21.7	14	0.7	1081	22.7	135	1.0	983	20.3	− 98	− 2.4
**Number of people with low trauma hip Fx + surgical Fx repair**														
Mar–June	605	58.9	650	59.3	45	0.5	656	61.1	6	1.8	615	60.9	− 41	− 0.2
June–Sept	574	61.6	573	60.6	− 1	− 1.0	675	62.4	102	1.9	631	64.2	− 44	1.8
**All Fx + surgical intervention < 24 h**														
Mar–June	761	74.0	819	74.7	58	0.7	807	75.1	− 12	0.4	754	74.7	− 53	− 0.5
June–Sept	701	75.2	728	77.0	27	1.7	822	76.0	94	− 0.9	750	76.3	− 72	0.3
**Hip Fx + surgical intervention < 24 h**														
Mar–June	573	94.7	624	96.0	51	1.3	627	95.6	3	− 0.4	587	95.4	− 40	− 0.1
June–Sept	543	94.6	553	96.5	10	1.9	649	96.1	96	− 0.4	600	95.1	− 49	− 1.1
**Deaths: all Fx + surgical Fx repair**														
Mar–June	55	5.4	51	4.7	− 4	− 0.7	51	4.8	0	0.1	41	4.1	− 10	− 0.7
June–Sept	47	5.0	36	3.8	− 11	− 1.2	54	5.0	18	1.2	30	3.1	− 24	− 1.9
**Deaths: hip Fx + surgical Fx repair**														
Mar–June	45	7.4	46	7.1	1	− 0.4	42	6.4	− 4	− 0.7	35	5.7	− 7	− 0.7
June–Sept	40	7.0	30	5.2	− 10	− 1.7	43	6.4	13	1.1	25	4.0	− 18	− 2.4
**Any complication post-surgical Fx repair**														
Mar–June	83	8.1	93	8.5	10	0.4	96	8.9	3	0.5	78	7.7	− 18	− 1.2
June–Sept	72	7.7	59	6.2	− 13	− 1.5	90	8.3	31	2.1	66	6.7	− 24	− 1.6
**Pneumonia**														
Mar–June	22	26.5	32	34.4	10	7.9	31	32.3	− 1	− 2.1	24	30.8	− 7	− 1.5
June–Sept	20	27.8	24	40.7	4	12.9	25	27.8	1	− 12.9	20	30.3	− 5	2.5
**Infection**														
Mar–June	19	22.9	22	23.7	3	0.8	20	20.8	− 2	− 2.8	12	15.4	− 8	− 5.5
June–Sept	14	19.4	13	22.0	− 1	2.6	30	33.3	17	11.3	22	33.3	− 8	0.0
**Other**														
Mar–June	51	61.5	49	52.7	− 2	− 8.8	51	53.1	2	0.4	46	59.0	− 5	5.9
June–Sept	43	59.7	30	50.9	− 13	− 8.9	42	46.7	12	− 4.2	32	48.5	− 10	1.8

Abbreviations: *Mar* March; *Sep* September; *Fx* fracture

^*^Low-energy fractures are defined as fractures sustained when falling from standing height or less

^†^The COVID-19 State of Public Health Emergency in Alberta resulted in a temporary residential lockdown, closure/restricted access of public facilities, and cancellation of elective surgeries (i.e., lockdown period)

^‡^The percent point change was calculated relative to the previous year (e.g., values from 2020 to 2019)

Across both the control and COVID-19 periods, patients with low-energy hip fractures accounted for the majority of low-energy fracture patients receiving surgery within the same episode of care (range: 58.9–64.2%). While the absolute number of surgeries decreased slightly during the COVID-19 periods relative to 2019 (March to June: 41 patients; June to September: 44 patients), these numbers are within the range observed during the control period (Fig. 4; Table 1).

Furthermore, patients with low-energy hip fractures made up the majority of patients receiving surgical repair within 24 h of being discharged from the ED both within the control period and in the COVID-19 period (range: 75.3–80.0%, data not reported). The proportion of hip fracture surgeries occurring within 24 h was high across the entire study period including COVID-19, ranging from 94.6 to 96.5%.

### Length of hospital stay

There was considerable variability in the mean length of hospital stay over the control period. Due to a skewed distribution, the median length of stay is presented. Overall, the median length of hospital stay remained stable across the control and COVID-19 study periods (Supplementary Table 2; Supplementary Fig. 1).

The median length of in-patient hospital stay did not change for all low-energy fractures over the March to September COVID-19 periods relative to the 2019 control period. However, the absolute number of days decreased by *n* = 1 day for the patients with hip fractures post-surgical fracture repair in March to June COVID-19 period relative to the 2019 control period (March to June 2019: 10.0 [IQR: 6.0–21.0] days vs. March to June COVID-19: 9.0 [IQR: 5.0–20.0] days). The standard deviation for the length of stay also decreased during the COVID-19 period from 48.1 to 26.0 in the March to June 2019 and COVID-19 periods, respectively, and from 50.8 to 28.4 in the June to September 2019 and COVID-19 periods, respectively, indicating less variability in the length of stay during the COVID-19 periods.

Of note, in-patient surgery for low-energy fractures was primarily composed of individuals with hip fractures. Nearly 100% of individuals with a hip fracture underwent surgery, and as mentioned previously, accounted for 58.9–64.2% of individuals receiving fracture repair surgery in the same episode of care.

### Death and complications

**Fig. 5:**
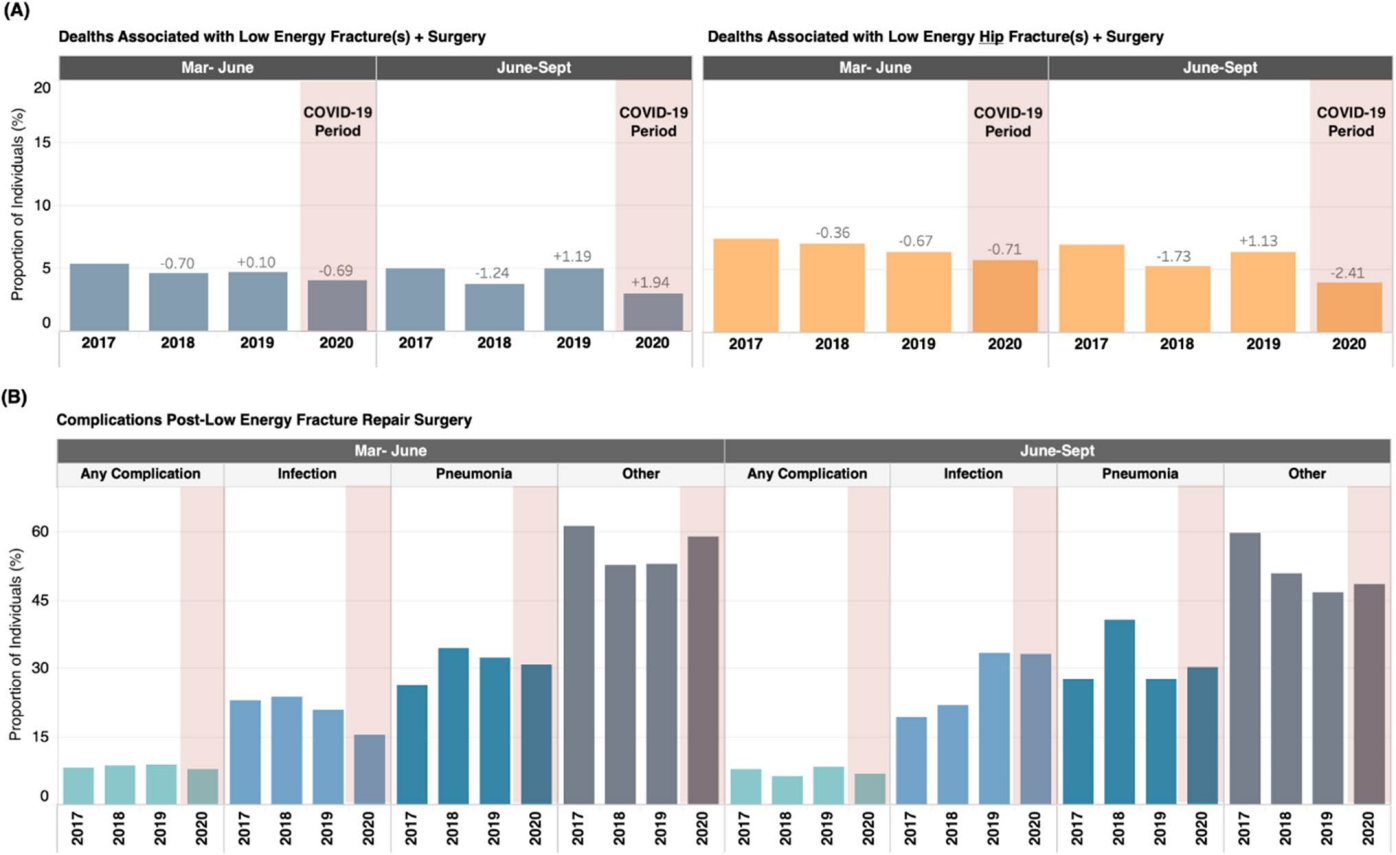
In-hospital deaths and complications post-fracture repair surgery, among patients with low-energy fractures who received surgery. (**A**) Proportion of patients with fracture diagnosis and surgical fracture repair codes within the same episode of care for all low-energy fractures* and low-energy hip fractures* who died in hospital following surgical fracture repair. (**B**) Proportion of patients with fracture diagnosis and surgical fracture repair codes within the same episode of care for all low-energy fractures* and low-energy hip fractures* who experienced complications post fracture repair. Complications are further stratified by type (infection, pneumonia, other). Abbreviations: Mar: March; Sep: September; *Low-energy fractures are defined as fractures sustained when falling from standing height or less

The absolute number of in-hospital patient deaths following surgical fracture repair of all fractures decreased by 10.0 and 24.0 deaths during the March to June and June to September COVID-19 periods, respectively, relative to the 2019 control periods (Fig. 5; Table 1). The observed decrease in the number of patient deaths amounted to a decrease of 0.7% percentage points (4.8% vs 4.1%) and 1.9% (5.0% vs 3.1%) points for the in-hospital mortality rate following surgery for March to June and June to September periods, respectively.

The observed decrease of in-hospital deaths following surgical repair of hip fracture patients (of whom account for over half of all low-energy fracture repair surgeries) likely contributed to the decrease in overall deaths (hip fracture: March to June COVID-19 vs. 2019: *n* =  − 7 deaths, a 0.7% point decrease in mortality rate among those who had hip fracture surgery; June to September: *n* =  − 18 deaths, a 2.4% point decrease). Patients with low-energy hip fractures accounted for the majority of deaths for all low-energy fractures receiving surgical intervention (approximately 80–90%, data not reported) on any given year.

The greatest decrease of in-hospital deaths for people with fracture undergoing surgical intervention was reported from June to September of the COVID-19 period for both all fractures (− 24 deaths) and hip fractures (− 18 deaths).

The trends in complications related to low-energy fractures varied during the COVID-19 period based on the type of complication. Overall, the proportion of patients with any complication following surgery was lower in the COVID-19 period (March to June: 7.7%; June to September: 6.7%) relative to the 2019 periods (March to June: 8.9%; June to September: 8.3%), but within the range observed across all the control years. The absolute number of patients experiencing complications following surgery decreased during the COVID-19 periods relative to the 2019 control period, across all types of complications of interest, likely because of the absolute number of patients receiving surgery. For pneumonia and “other” complications (e.g., myocardial infarction, venous thromboembolism), the proportion of patients experiencing these complications during the COVID-19 periods was within the range observed in the control periods. However, the proportion of patients experiencing infection complications was lower during the March to June COVID-19 period (15.4% of patients who had surgery) relative to any of the control periods (range: 20.8–23.7%).

## Discussion

The results of this study show that low-energy fractures remained a public health concern over the lockdown period, accounting for a large majority of overall fractures. The majority of low-energy fractures in this population often occur either spontaneously (e.g., compression fractures of the spine) or by sustaining a low-energy fall (e.g., at home or outdoors) from standing height or less [20]. Data suggest that the first state of emergency period within Alberta had a slight impact on the total number of fractures recorded (both overall and low-energy fractures), with vertebral, hip, and proximal/upper humerus fractures all experiencing slight declines in the March to June 2020 period, relative to previous years. The number of patients with fractures increased in the June to September 2020 period. Drivers of these changes may include decreased movement and activity resulting from the stay-at-home public health orders in the March to June 2020 period and fear or hesitation to seek medical attention at an in-hospital setting during this lockdown period. As the lockdown was lifted, increased activity (thus subsequent risk of falling) and health-seeking behavior may contribute to the increase in observed fractures. A depletion-of-susceptible individuals bias is not anticipated to be a driver of the reduced fractures observed in the first COVID-19 period, as the population ≥ 50 years of age including among those ≥ 80 years of age has continued to grow year over year in Alberta [21] and the excess mortality was fairly comparable between the two COVID periods [22].

Increased time to surgery following fracture is known to be associated with increased mortality, particularly for hip fractures [23, 24]. Although these results show a decrease in the absolute number of patients who received surgical intervention for fracture repair in less than 24 h, the proportion of patients who received surgery in less than 24 h, including those with a hip fracture, remained relatively stable relative to the previous year. During lockdown, elective surgeries were delayed in the first period of the pandemic to provide additional room for patients presenting to the hospital with COVID-19. These data suggest that the cancellation of elective surgeries did not influence the timing of surgery for this population. The number of hospitalized COVID cases did not exceed hospital capacity during the first wave in Alberta, likely with minimal impact to operating room access.

The results suggest that the median length of stay in-hospital for all low-energy fractures and low-energy hip fractures remained consistent. Medians for all fractures and hip fractures did not change with the exception of hip fractures from March to June of the COVID-19 period which was reduced by 1 day. Length of hospital stay results should be interpreted with caution considering the large variability of length of stay data demonstrated by the interquartile ranges for each study period. Although small, the decreased length of stay during the March to June COVID-19 period relative to the previous year may be reflective of patients being discharged early to create space for critically ill patients with COVID-19 infection. The large standard deviation observed in the length of stay data suggests that there are patients with exceptionally long length of stay, possibly awaiting an alternative level of care rather than an acute stay. The standard deviation was much smaller during the COVID-19 period possibly due to changes in care for such patients [25]. Another challenge during the pandemic was care facilities being put on outbreak status and preventing patients from being transferred back to their long-term care homes. Many nursing homes were not accepting new patients during the lockdown, which resulted in hospitals creating space on another ward for patients to await transfer, potentially increasing the time that a patient would stay in the hospital [26, 27].

Early data also suggest an overall decrease in the absolute number of complications following surgical intervention for low trauma fracture, in line with the lower total number of patients with fractures. The proportion of patients experiencing complications during the COVID-19 periods was within range of the control periods for all complications except post-surgical infections, which had a decrease during the March to June COVID-19 period relative to the 2019 control period. It is difficult to ascertain the reason for this observed decrease; however, increased sanitation practices and potential changes to exposure protocols in hospitals during lockdown may have been contributing factors.

The absolute number of deaths post-surgical fracture repair decreased over the first two periods of the COVID-19 lockdown, largely driven by hip fractures which accounted for more than half of the low-energy fractures receiving surgical repair in this population. The greatest decrease in deaths post-surgical fracture repair was observed in the June to September COVID-19 period relative to the 2019 control period. The decrease in complications observed during the March to June COVID-19 period may have contributed to a decrease in death during the June to September COVID-19 period relative to the previous year. Alternatively, the decrease in death could also be associated with the slight decrease observed in the length of stay during the COVID-19 period. Patient death was captured as occurring in-hospital, as such, death following discharge would not have been captured in this study. Of note, there was an overall increase in all-cause deaths in the province [28]. Alberta had an excess of 682 deaths from March to September of the COVID-19 period, relative to previous years [22]. Therefore, the deaths reported herein may not represent the totality of deaths associated with a high mortality fracture (e.g., hip fracture) that could have taken place following the transfer of a patient from the hospital to home, a rehabilitation facility, or a long-term care home, or among patient that did not seek care for a fracture. There were also no homecare physiotherapy, osteoporosis physiotherapy programs, or private clinics open during the first lockdown, possibly increasing the number of deaths related to fractures following interaction in a hospital setting [29].

### Strengths and limitations

To our knowledge, this is the first population-based Canadian study to evaluate the immediate impact of the early COVID-19 pandemic lockdown on acute FF care within an Alberta population. Strengths of this data include a 3-year control period to capture overall variability as well as seasonal fluctuations in fracture rates. This study gathered data from population-level provincial databases to provide a comprehensive, descriptive analysis of the demographic most at risk for FF whose care may have been impacted by the first COVID lockdown.

The data reported herein is open year administrative health data. At the time of data receipt, open year data had not been finalized but are considered to be complete (i.e., additional records are not submitted after the mandatory 3-month reporting period). Assumptions have been made to isolate the population of interest. Data were captured to identify anyone in Alberta ≥ 50 years of age, who are considered to be at a higher risk for osteoporosis, reporting to the hospital with a fracture. However, a limitation of this data is that the diagnosis or severity of osteoporosis could not be confirmed. For the surgical analysis, it was assumed that individuals who had received a fracture diagnosis code and a pre-specified surgical fracture repair code within the same episode of care had received surgical repair for their fracture. However, surgical repair of the fracture and/or a potentially unrelated surgery within that episode of care were not confirmed. Lastly, the study design is cross-sectional in nature and only captures a short period of time in which the frequency of fractures was assessed. As mentioned previously, the utilization of a 3-year control period to assess potential changes in rates for outcomes related to fracture during the COVID-19 lockdown period strengthens the study design; however, inferences should be interpreted with caution. Additionally, the health system was not at capacity during the COVID-19 period assessed in this study; therefore, external generalizability may be limited.

### Future directions

Future studies should aim to better understand longitudinal outcomes for patients sustaining fracture during the COVID-19 pandemic. An attempt to capture any changes in treatment patterns that took place during various lockdown periods may provide data that will be invaluable to the assessment of subsequent fractures, morbidity, mortality, and the economic burden associated with this population. Importantly, it is unknown at this time if the continuity of care demonstrated in the initial stages of the pandemic, reported here, was sustained throughout the pandemic. Subsequent waves of the pandemic in Alberta were characterized by substantial increases in hospitalized COVID cases that exceeded hospital capacity. Continued analysis of administrative data throughout the duration of the pandemic would be beneficial as the demands on the health care system changed substantially in Alberta across subsequent COVID-19 waves, all of which are anticipated to have major impacts on the management of osteoporosis as well as other diseases.

## Conclusion

The current results provide descriptive evidence of low-energy fracture frequency and acute low-energy fracture management in Alberta, Canada, during the onset (first wave) of the COVID-19 pandemic. Data suggest that low-energy fracture rates declined slightly but largely remained stable in the first few months of the pandemic. This is an important finding as over half of the patients ≥ 50 years of age presenting to a hospital across both the control and COVID-19 periods with suspected low-energy FF were hip fracture patients that required surgery.

Although early results suggest little to no change in the care of low-energy fractures, particularly low trauma hip fractures requiring surgical repair, this may not accurately reflect acute treatment of low-energy fractures during subsequent COVID-19 waves when the healthcare system was under severe strain. Further investigation regarding acute care and treatment patterns throughout the course of the COVID-19 pandemic is warranted.

## Supplementary Information

Below is the link to the electronic supplementary material.

Supplementary file1 (DOCX 163 KB)

## Data Availability

Data was obtained via data request to Alberta Health.

## Code availability

Study methodology documentation and statistical programming code used in this study are available upon request.
