# Supplementary material for: Trends in osteoporotic fracture and related in-hospital complications during the COVID-19 pandemic in Alberta, Canada
Source: Arch Osteoporos. 2022 Aug 3;17(1):109. doi: 10.1007/s11657-022-01114-9 (PMC9349109; doi:10.1007/s11657-022-01114-9)
Supplement: Supplementary file 1 — Supplementary file1 (DOCX 163 KB) [file 11657_2022_1114_MOESM1_ESM.docx]

**Trends in Osteoporotic Fracture and Related In-Hospital Complications during the COVID-19 pandemic in Alberta, Canada**

T. Oliveira, D.L. Kendler, P. Schneider, A.G. Juby, R.J. Wani, M. Packalen, S. Avcil, S. Li, C. Waters-Banker, E. Graves, S. McMullen, J. Brown

**Supplemental Materials**

**Prepared for: Osteoporosis International**

**Corresponding Author: Suzanne McMullen**

**Medlior Health Outcomes Research Ltd.**

**Suite 210 – 28 Quarry Park Blvd**

**Calgary, AB**

**T2C 5P9**

**Tel: 604-250-7497**

**Suzanne.mcmullen@medlior.com**

**Date: 11 April 2022**

Supplementary Table 1: Absolute Number of Patients with Low-energy Fracture(s)* Reported by Period, Stratified by Age and Sex in Alberta, Canada (2017-2020)

|  | **2017** | | **2018** | | **Difference (2018-2017)** | | **2019** | | **Difference (2019-2018)**^‡^ | | **2020**  **COVID-19**^†^ | | **Difference (2020-2019)**^‡^ | |
| --- | --- | --- | --- | --- | --- | --- | --- | --- | --- | --- | --- | --- | --- | --- |
| **Number of People with Low Trauma Fracture(s)** | n | % | n | % | n | % Point  Change^‡^ | n | % | n | % Point  Change^‡^ | n | % | n | % Point  Change^‡^ |
| Mar – June | 4,531 | - | 4,884 | - | 353 | - | 4,784 | - | -100 | - | 4,308 | - | -476 | - |
| June – Sept | 4,438 | - | 4,361 | - | -77 | - | 4,761 | - | 400 | - | 4,831 | - | 70 | - |
| **50-64 Years** | | | | | | | | | | | | | | |
| *Females* |  |  |  |  |  |  |  |  |  |  |  |  |  |  |
| Mar – June | 934 | 20.6 | 1,080 | 22.1 | 146 | 1.5 | 935 | 19.5 | -145 | -2.6 | 877 | 20.4 | -58 | 0.8 |
| June – Sept | 888 | 20.0 | 824 | 18.9 | -64 | -1.1 | 900 | 18.9 | 76 | 0.0 | 935 | 19.4 | 35 | 0.5 |
| *Males* |  |  |  |  |  |  |  |  |  |  |  |  |  |  |
| Mar – June | 522 | 11.5 | 542 | 11.1 | 20 | -0.4 | 500 | 10.5 | -42 | -0.6 | 455 | 10.6 | -45 | 0.1 |
| June – Sept | 467 | 10.5 | 446 | 10.2 | -21 | -0.3 | 461 | 9.7 | 15 | -0.5 | 473 | 9.8 | 12 | 0.1 |
| **65-79 Years** | | | | | | | | | | | | | | |
| *Females* |  |  |  |  |  |  |  |  |  |  |  |  |  |  |
| Mar – June | 1,032 | 22.8 | 1,123 | 23.0 | 91 | 0.2 | 1146 | 24.0 | 23 | 1.0 | 1,016 | 23.6 | -130 | -0.4 |
| June – Sept | 1,009 | 22.7 | 1,050 | 24.1 | 41 | 1.3 | 1173 | 24.6 | 123 | 0.6 | 1,216 | 25.2 | 43 | 0.5 |
| *Males* |  |  |  |  |  |  |  |  |  |  |  |  |  |  |
| Mar – June | 461 | 10.2 | 526 | 10.8 | 65 | 0.6 | 521 | 10.9 | -5 | 0.1 | 510 | 11.8 | -11 | 0.9 |
| June – Sept | 466 | 10.5 | 445 | 10.2 | -21 | -0.3 | 547 | 11.5 | 102 | 1.3 | 563 | 11.7 | 16 | 0.2 |
| **80+ Years** | | | | | | | | | | | | | | |
| *Females* |  |  |  |  |  |  |  |  |  |  |  |  |  |  |
| Mar – June | 1,159 | 25.6 | 1,181 | 24.2 | 22 | -1.4 | 1,198 | 25.0 | 17 | 0.9 | 1,059 | 24.6 | -139 | -0.5 |
| June – Sept | 1,253 | 28.2 | 1,177 | 27.0 | -76 | -1.2 | 1,270 | 26.7 | 93 | -0.3 | 1,231 | 25.5 | -39 | -1.2 |
| *Males* |  |  |  |  |  |  |  |  |  |  |  |  |  |  |
| Mar – June | 423 | 9.3 | 432 | 8.8 | 9 | -0.5 | 484 | 10.1 | 52 | 1.3 | 391 | 9.1 | -93 | -1.0 |
| June – Sept | 355 | 8.0 | 419 | 9.6 | 64 | 1.6 | 410 | 8.6 | -9 | -1.0 | 413 | 8.5 | 3 | -0.1 |

Abbreviations: COVID-19: coronavirus disease 2019 SARS-CoV-2 virus; Mar: March; Jun: June; Sep: September; Dec: December

*Low-energy fractures are defined as fractures sustained when falling from standing height or less.

†The COVID-19 State of Public Health Emergency in Alberta resulted in the temporary residential lockdown and closure/restricted access of public facilities

‡The absolute difference was calculated relative to the previous year

Supplementary Table 2: Median Length of Hospital Stay for Patients with Low-Energy* Fracture Diagnosis and Surgical Repair Codes by Study Period in Alberta, Canada (2017 – 2020)

|  | **2017** | | **2018** | | **Difference**  **(2018-2017)** | | **2019** | | **Difference**  **(2019-2018)** | | **2020 COVID-19**  **Lockdown**^†^ | | **Difference**  **(2020-2019)** | | |
| --- | --- | --- | --- | --- | --- | --- | --- | --- | --- | --- | --- | --- | --- | --- | --- |
|  | Median | IQR | Median | IQR | Change in Median^‡^ | % Change^‡^ | Median | IQR | Change in Median^‡^ | % Change^‡^ | Median | IQR | Change in Median^‡^ | % Change^‡^ |  |
| **All Fractures Median LoS** |  |  |  |  |  |  |  |  |  |  |  |  |  |  |  |
| Mar – June | 9 | 5.0-19.0 | 8 | 5.0-15.0 | -1.0 | -0.1 | 9 | 5.0-19.0 | 1.0 | 0.1 | 9 | 4.0-19.0 | 0.0 | 0.0 |  |
| June - Sept | 9 | 5.0-16.0 | 9 | 5.0-17.0 | 0.0 | 0.0 | 9 | 5.0-20.0 | 0.0 | 0.0 | 9 | 5.0-19.0 | 0.0 | 0.0 |  |
| **Hip Fractures Median LoS** |  |  |  |  |  |  |  |  |  |  |  |  |  |  |  |
| Mar – June | 10 | 6.0-21.0 | 9 | 6.0-16.0 | -1.0 | -0.1 | 10 | 6.0-21.0 | 1.0 | 0.1 | 9 | 5.0-20.0 | -1.0 | -10.0 |  |
| June - Sept | 9 | 6.0-16.0 | 9 | 5.0-17.0 | 0.0 | 0.0 | 10 | 6.0-20.0 | 1.0 | 0.1 | 10 | 6.0-20.0 | 0.0 | 0.00 |  |

Abbreviations: Mar: March; Jun: June; Sep: September; Dec: December; Fx: Fracture

*Low-energy fractures are defined as fractures sustained when falling from standing height or less.

†The COVID-19 State of Public Health Emergency in Alberta resulted in a temporary residential lockdown, closure/restricted access of public facilities, and cancellation of elective surgeries (i.e., lockdown period).

‡Absolute change was calculated relative from the previous year (e.g., 2020-2019). The percent change in rate was calculated relative to the previous year (e.g., values from 2020-2019 ÷ 2019)


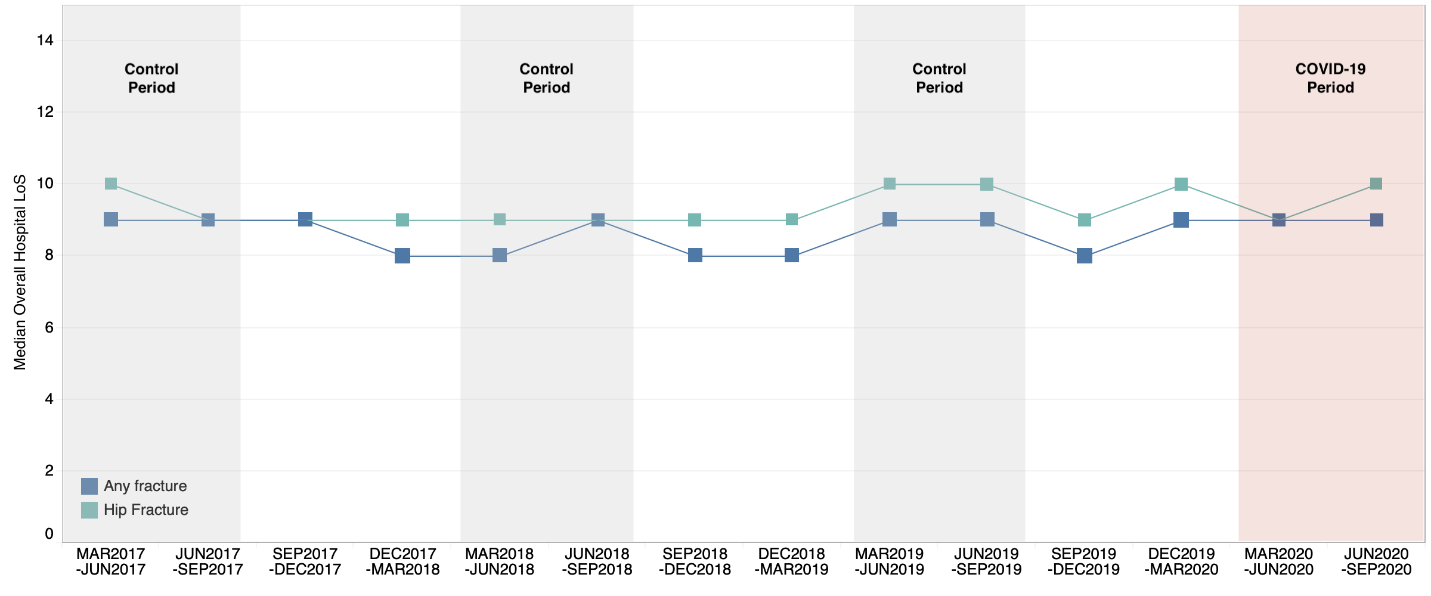


Supplementary Fig 1: Median length of stay in hospital for ‘all low-energy fractures*’ (blue) and ‘low-energy hip fractures*’ (green) in Alberta, Canada (2017–2020).

**Note:** The grey shaded areas represent the control periods evaluated in this study. The red shaded area represents the COVID-19 pandemic period where in the first three months (Mar-Jun) a COVID-19 State of Public Health Emergency in Alberta was active, which resulted in a temporary residential lockdown, the closure/restricted access of public facilities, and the cancellation of elective surgeries (i.e., lockdown period).

Abbreviations: LoS: length of stay; Mar: March; Jun: June; Sep: September; Dec: December

*Low-energy fractures are defined as fractures sustained when falling from standing height or less.
